# Changes in Physical Activity Following a Genetic-Based Internet-Delivered Personalized Intervention: Randomized Controlled Trial (Food4Me)

**DOI:** 10.2196/jmir.5198

**Published:** 2016-02-05

**Authors:** Cyril FM Marsaux, Carlos Celis-Morales, Katherine M Livingstone, Rosalind Fallaize, Silvia Kolossa, Jacqueline Hallmann, Rodrigo San-Cristobal, Santiago Navas-Carretero, Clare B O'Donovan, Clara Woolhead, Hannah Forster, George Moschonis, Christina-Paulina Lambrinou, Agnieszka Surwillo, Magdalena Godlewska, Jettie Hoonhout, Annelies Goris, Anna L Macready, Marianne C Walsh, Eileen R Gibney, Lorraine Brennan, Yannis Manios, Iwona Traczyk, Christian A Drevon, Julie A Lovegrove, J Alfredo Martinez, Hannelore Daniel, Michael J Gibney, John C Mathers, Wim HM Saris

**Affiliations:** ^1^ Department of Human Biology NUTRIM School of Nutrition and Translational Research in Metabolism Maastricht University Medical Centre + (MUMC+) Maastricht Netherlands; ^2^ Human Nutrition Research Centre Institute of Cellular Medicine Newcastle University Newcastle Upon Tyne United Kingdom; ^3^ Hugh Sinclair Unit of Human Nutrition and Institute for Cardiovascular and Metabolic Research University of Reading Reading United Kingdom; ^4^ Zentralinstitut für Ernährungs- und Lebensmittelforschung (ZIEL) Research Center of Nutrition and Food Sciences Biochemistry Unit Technische Universität München München Germany; ^5^ Department of Nutrition, Food Science and Physiology Centre for Nutrition Research University of Navarra Pamplona Spain; ^6^ Centro de Investigación Biomédica en Red-Fisiopatología de la Obesidad y Nutrición (CIBERobn) Instituto de Salud Carlos III Madrid Spain; ^7^ University College Dublin (UCD) Institute of Food and Health University College Dublin Belfield Dublin Ireland; ^8^ Department of Nutrition and Dietetics Harokopio University Athens Greece; ^9^ National Food & Nutrition Institute (IZZ) Warsaw Poland; ^10^ Experiences Research Department Philips Research Eindhoven Netherlands; ^11^ Personal Health Solutions Philips Consumer Lifestyle Amsterdam Netherlands; ^12^ Department of Nutrition, Institute of Basic Medical Sciences Faculty of Medicine University of Oslo Oslo Norway

**Keywords:** *FTO*, physical activity, personalized intervention, randomized controlled trial, genetic testing, disclosure, behavior change, Web based

## Abstract

**Background:**

There is evidence that physical activity (PA) can attenuate the influence of the fat mass- and obesity-associated (*FTO*) genotype on the risk to develop obesity. However, whether providing personalized information on *FTO* genotype leads to changes in PA is unknown.

**Objective:**

The purpose of this study was to determine if disclosing *FTO* risk had an impact on change in PA following a 6-month intervention.

**Methods:**

The single nucleotide polymorphism (SNP) *rs9939609* in the FTO gene was genotyped in 1279 participants of the Food4Me study, a four-arm, Web-based randomized controlled trial (RCT) in 7 European countries on the effects of personalized advice on nutrition and PA. PA was measured objectively using a TracmorD accelerometer and was self-reported using the Baecke questionnaire at baseline and 6 months. Differences in baseline PA variables between risk (AA and AT genotypes) and nonrisk (TT genotype) carriers were tested using multiple linear regression. Impact of *FTO* risk disclosure on PA change at 6 months was assessed among participants with inadequate PA, by including an interaction term in the model: disclosure (yes/no) × *FTO* risk (yes/no).

**Results:**

At baseline, data on PA were available for 874 and 405 participants with the risk and nonrisk *FTO* genotypes, respectively. There were no significant differences in objectively measured or self-reported baseline PA between risk and nonrisk carriers. A total of 807 (72.05%) of the participants out of 1120 in the personalized groups were encouraged to increase PA at baseline. Knowledge of *FTO* risk had no impact on PA in either risk or nonrisk carriers after the 6-month intervention. Attrition was higher in nonrisk participants for whom genotype was disclosed (*P*=.01) compared with their at-risk counterparts.

**Conclusions:**

No association between baseline PA and *FTO* risk genotype was observed. There was no added benefit of disclosing *FTO* risk on changes in PA in this personalized intervention. Further RCT studies are warranted to confirm whether disclosure of nonrisk genetic test results has adverse effects on engagement in behavior change.

**Trial Registration:**

ClinicalTrials.gov NCT01530139; http://clinicaltrials.gov/show/NCT01530139 (Archived by WebCite at: http://www.webcitation.org/6XII1QwHz)

## Introduction

The prevalence of physical inactivity in Europe and worldwide is high [1]. Given that physical inactivity is among the top risk factors for noncommunicable diseases [2], finding effective ways to achieve long-lasting improvements in physical activity (PA) remains a major challenge [3]. While previous intervention strategies have mainly focused on a "one-size-fits-all" approach to change behavior, recent studies have used personalized approaches, such as tailored Web-based interventions [4,5]. There is inconsistent evidence on whether these personalized approaches are more effective at increasing PA than standard guidelines, and effects, when present, are often small and with short-term efficacy [6]. Concurrently, there has been a growing interest in using genetic information to personalize lifestyle interventions [7]. Although disclosure of such information does not appear to have unintended adverse effects, more randomized controlled trials (RCTs) are needed to establish whether gene-based personalized interventions promote greater behavior change than conventional "one-size-fits-all" interventions [8]. In particular, data on whether providing genetic information leads to an increase in PA are lacking.

The fat mass- and obesity-associated (*FTO*) gene has provided strong evidence of the genetic susceptibility to obesity. Polymorphisms in this gene located in intron 1 and exon 2 have been shown to be consistently and strongly associated with obesity-related markers [9,10]. For instance, individuals homozygous for the higher-risk allele, AA, of single nucleotide polymorphism (SNP) rs9939609 in the FTO gene *FTO* weighed, on average, 3 kg more and had 1.7-fold increased odds of having obesity compared with those homozygous for the lower-risk allele, TT [11]. Moreover, there is increasing evidence that the *FTO* genetic susceptibility to obesity can be modulated by lifestyle factors, and that PA, for example, may attenuate the effects of the *FTO* genotype on obesity-related traits [12-17]. However, to our knowledge there is no data on whether disclosing information on *FTO* genotype can motivate individuals to increase their PA. Elucidating whether genetic-based advice can promote improvements in PA behaviors may help in the design of more effective interventions, especially when tailored to individuals who would benefit most from increasing their PA.

As part of the Food4Me study (ClinicalTrials.gov number: NCT01530139)—a Web-based RCT in 7 European countries—we investigated the effects of 3 levels of personalized advice on changes in PA, including a level with genetic information on *FTO* [18,19]. See Multimedia Appendix 1 for the CONSORT-EHEALTH checklist [20]. We found that personalized feedback in general led to greater improvements in self-reported PA, but not in objectively measured PA, compared with standard guidelines [19]. However, we did not investigate the effect of disclosing genetic-based information on PA change, and whether the response differs between carriers of a genetic risk and nonrisk carriers. Thus, the aim of these analyses was to assess the impact of knowledge of *FTO* risk status on change in self-reported and objectively measured PA in Food4Me participants.

## Methods

### Subjects

Subjects were participants of the Food4Me study, a 6-month, Web-based RCT on personalized nutrition and lifestyle conducted in 7 European countries—Germany, Greece, Ireland, the Netherlands, Poland, Spain, and the United Kingdom. As outlined elsewhere [18], 1607 adults aged ≥18 years were randomized to the study. Exclusion criteria included no or limited access to the Internet, following a prescribed diet, or having altered nutritional requirements because of a medical condition. The local ethics committee of each recruiting center approved the study protocol and all subjects provided informed consent digitally before participating.

### Study Design

Participants were randomly allocated to one of the 4 groups—Level 0: standard, nonpersonalized, dietary and PA guidelines; Level 1: dietary and PA advice based on current diet and PA; Level 2: dietary and PA advice based on current diet, PA, and phenotype (eg, waist circumference and blood cholesterol); and Level 3: dietary and PA advice based on current diet, PA, phenotype, and genotype (eg, *FTO*). The randomization scheme has been described previously [18]. All data were collected remotely following standardized operating procedures. At baseline, participants received study kits by post containing all necessary materials, such as an accelerometer and DNA collection kit (see the Physical Activity Assessment and Genotyping sections below), to perform measurements at home, but used their own scales to measure body weight. Printed instructions were included and demonstration videos were available on the Food4Me website [18,21].

On the allocated study day and following an 8-hour overnight fast, participants collected a buccal cell sample for DNA; measured their height, weight, and waist circumference; and started wearing an accelerometer. The buccal cell sample was returned to the research center in a prepaid stamped addressed envelope and anthropometric measurement values were self-reported online. Questionnaires to be completed online the same day included the Baecke PA questionnaire (see the Physical Activity Assessment section below). Participants repeated the measurements, except DNA collection, at 3 and 6 months [18].

Following measurements at baseline and 3 months, participants received, at both time points, a personalized (Levels 1-3) or nonpersonalized (Level 0) report, including feedback on PA according to their group. The personalized feedback provided was based on a predefined set of algorithms, including anthropometric, PA (Levels 1-3), phenotypic (Levels 2 and 3), and genotypic (Level 3 only) data. Results in the personalized report were compared with recommendations for each anthropometric, PA (Levels 1-3), and phenotypic (Levels 2 and 3) item, using 3-color graded lines—green: good; amber: improvement recommended; and red: improvement strongly recommended. In addition, Level 3 participants received information in their report about 5 diet- and lifestyle-related genes [18]. For *FTO*, the message was “A specific variation of this gene is associated with a greater need to maintain a healthy body weight and engage in physical activity. A healthy weight combined with exercise may provide added health benefits for these individuals.” Participants were informed whether they were carriers of the *risk* variant for the *FTO* SNP rs9939609 (*yes* or *no*, if they were genotyped AA or AT, or TT, respectively). Each personalized report (Levels 1-3) also contained a specific message related to body weight and PA. Additionally, for Level 3 participants this specific message referred to *FTO*. For example, for an AA/AT participant with increased body mass index (BMI), increased waist circumference, and low PA, the message was “We recommend reducing your body weight and waist circumference to a healthy normal range because you have a genetic variation that can benefit by reducing these 2 obesity markers. Also, your physical activity level is too low.” Full details of the study design have been published elsewhere [18].

### Physical Activity Assessment

#### Objective Physical Activity

PA was assessed objectively using the TracmorD triaxial accelerometer (Philips Consumer Lifestyle, the Netherlands) [22,23]. Participants were instructed to wear the accelerometer every day while awake, except when taking a shower, for the entire duration of the 6-month study. Participants uploaded data every 2 weeks onto the study server via the Internet. Data were recorded with a time-sampling interval of 1 min. A day was considered valid if the participant had worn the TracmorD accelerometer between 10 and 18 h. Wear time was defined as 24 h minus nonwear time. To define nonwear time, we adapted the recommendations of Choi et al [24] to the TracmorD accelerometer. R software version 3.1.2 (The R Foundation) [25] was used for PA data processing.

Daily PA level (PAL)—the ratio of total energy expenditure to basal metabolic rate—was derived from activity counts [22]. Time spent in sedentary behavior—corresponding to <1.5 metabolic equivalents (METs)—and moderate- and vigorous-intensity PA—3 to <6 METs and ≥6 METs, respectively—were calculated based on the application of thresholds for activity energy expenditure (AEE) equivalent to the METs thresholds. Daily AEE was calculated as follows:

Daily AEE = (0.9 × daily PAL - 1) × BMR (1)

where the daily basal metabolic rate (BMR) is estimated using the Oxford equations developed by Henry, based on sex, age, and weight [26].

PA estimates were calculated over a 2-week period at baseline and 6 months. This 2-week assessment period occurred before any feedback was given for the corresponding time point. Sufficient PA data at each time point was defined as having at least 3 valid weekdays and 2 valid weekend days of accelerometer wear during the 2-week period. For individuals with sufficient PA data, mean data per day were calculated based on all valid week and weekend days of the assessment period as follows:

Mean = (mean for weekdays × 5 + mean for weekend days × 2) / 7 (2).

For sedentary time and time spent in moderate PA and vigorous PA, weekly estimates were calculated as follows:

Mean = (mean for weekdays × 5 + mean for weekend days × 2) (3).

#### Self-Reported Physical Activity

At each time point, participants completed the Baecke questionnaire online [27] based on their PA during the last month. This short, extensively validated questionnaire [28-30] is composed of 3 sections—work, sport, and nonsport leisure—with indices ranging from 1 to 5 and a sum total (ie, total activity index) ranging from 3 to 15. Scores were calculated at baseline and month 6, according to the questionnaire protocol [27].

### Genotyping

Participants collected a buccal cell sample at baseline, using Isohelix SK-1 DNA buccal swabs and Isohelix Dri-capsules (LGC Genomics, Hertfordshire, UK). Samples were returned to the recruiting centers and shipped to LGC Genomics, who extracted the DNA and used competitive allele-specific polymerase chain reaction (KASP) genotyping assays to provide biallelic scoring of SNP rs9939609 in the *FTO* gene.

### Statistical Analyses

Data are presented as means (SD) for continuous variables and as percentages for categorical variables, unless otherwise stated. A chi-square test was used to test if the observed *FTO* genotype counts were in Hardy-Weinberg equilibrium [31]. To examine if there was an association between PA and *FTO* genotype, we used baseline data and robust multiple linear regression models, based on computation of SMDM estimates [32] to account for violation of the normality assumption. *FTO* genotype was operationalized as *risk* (AA and AT) and *nonrisk* (TT).

To study the impact of knowledge of *FTO* risk status on changes in PA, we used two approaches. In the first approach or primary analysis, we investigated whether personalized advice based on genetic information (ie, *FTO* risk) was more effective at increasing PA than personalized advice without genetic-based information in *FTO* risk and nonrisk carriers. In this analysis, we compared Level 3 participants who received personalized advice to increase PA, including disclosure of *FTO* risk, with participants who received personalized advice to increase PA without any genetic-based information (pooled Levels 1 and 2). As a secondary analysis, we assessed whether personalized advice based on genetic information (ie, *FTO* risk) was more effective at increasing PA than standard guidelines (ie, nonpersonalized advice) in *FTO* risk and nonrisk carriers. This analysis compared Level 3 participants with the control group—Level 0, nonpersonalized guidelines. In order to match the characteristics of both groups, participants in the control group were included only if they had insufficient baseline PA (ie, they would have been advised to increase their PA if they had not been in the control group). For both primary and secondary analyses, we used robust multiple regression models, including an interaction term between *FTO* risk (*yes* or *no*) and disclosure of genetic information (*yes* or *no*). If there was no significant interaction, we looked at the main effects after removing the interaction term from the model. Models were adjusted for age, sex, country, BMI, season, accelerometer wear time, and baseline PA variable as appropriate. Additional sensitivity analyses were run, stratifying by sex and by tertile of baseline PA variables. Attrition rates between groups were compared using Pearson’s chi-square tests. R software version 3.1.2 (The R Foundation) [25] was used to perform all analyses and the significance level was set at *P*<.05.

## Results

### Attrition Rate and Compliance

**Figure 1:**
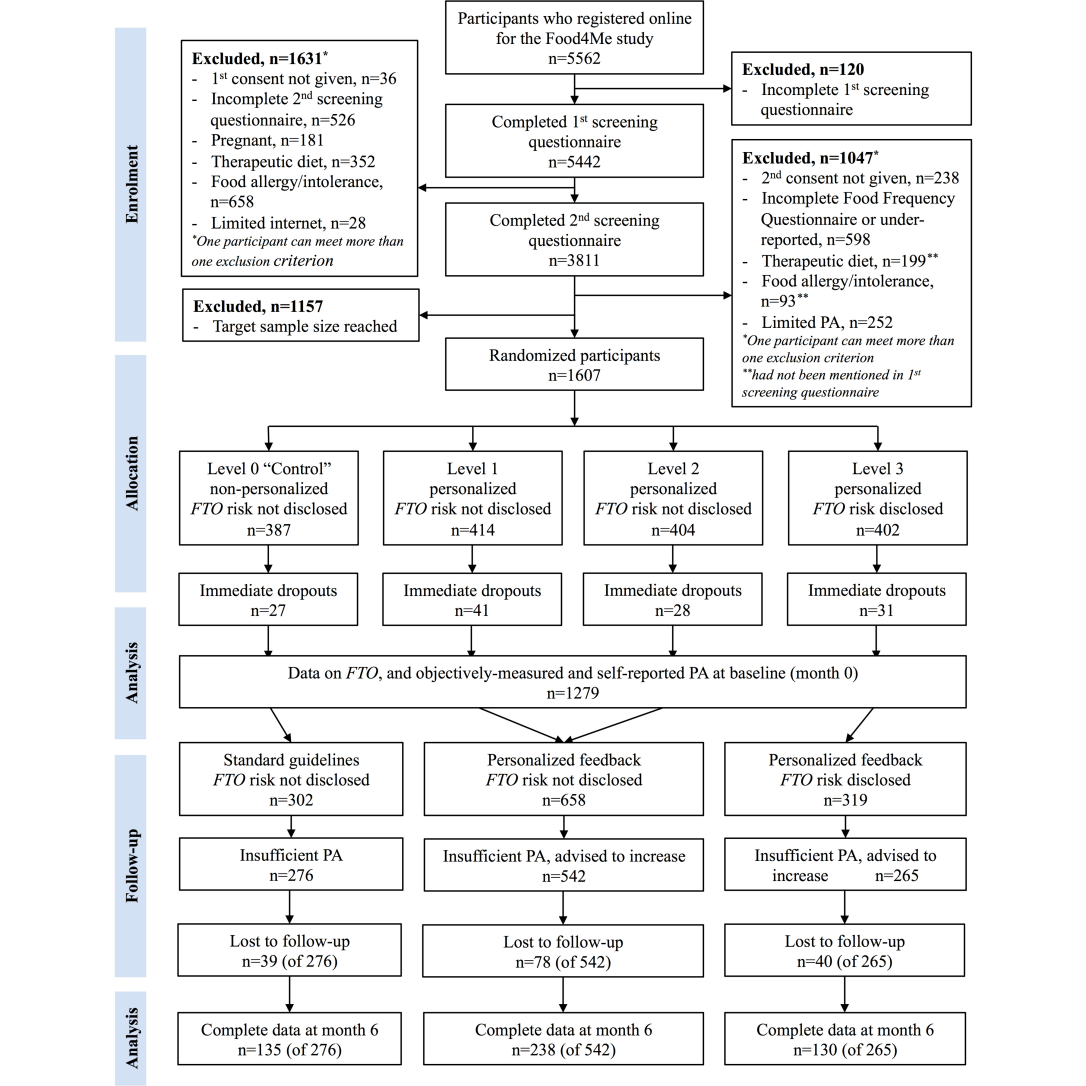
Flowchart of study procedures. Participants in Level 0 (controls) received standard, nonpersonalized guidelines during the intervention, whereas participants in Levels 1-3 received personalized advice. PA: physical activity; FTO: fat mass- and obesity-associated gene.

A total of 1607 individuals were randomized into the study (see Figure 1) and 127 (7.90%) of them dropped out before starting the trial; their characteristics will be reported elsewhere. Genotype and PA data were available for 1279 of the 1480 (86.42%) starters, which were therefore included in the baseline analysis (see Figure 1). Although sufficient accelerometer data were defined as having a minimum of 3 valid weekdays and 2 valid weekend days of accelerometer wear, 77.56% (992/1279) of subjects had 10 or more valid days of accelerometer wear at baseline—mean 11.3 days (SD 2.4): 8.2 weekdays (SD 1.9) and 3.2 weekend days (SD 0.8).

Among the 1120 participants who received personalized advice (Levels 1-3), 807 (72.05%) were advised to increase their PA following assessment of baseline PA. Similarly, in the control group (Level 0), 276 of 360 (76.7%) participants would have been advised to increase their PA if the algorithms applied to Levels 1-3 had been applied to the control group (see Figure 1). For these participants with inadequate PA, attrition rate was similar between groups (14-15%, *P*=.45) at month 6 (see Figure 1). In the group where *FTO* risk was disclosed (Level 3), participants with the nonrisk (TT) genotype were more likely to drop out of the intervention than the at-risk (AA/AT) participants—attrition rate 22% (TT) versus 12% (AA/AT); odds ratio (OR) 2.04, 95% CI 0.96-4.29, *P*=.04. This was also the case when considering all participants in Level 3 (ie, not only those advised to increase their PA)—attrition rate 20% (TT) versus 11% (AA/AT); OR 2.17, 95% CI 1.13-4.17, *P*=.01 (see Table 1). There were no significant differences in attrition rates after 6 months between risk and nonrisk carriers in any other groups.

Although only 157 out of 1083 (14.50%) participants with inadequate PA had dropped out by month 6, compliance with wearing the accelerometer decreased during the study. Thus, 46.45% (503/1083) of subjects had data on *FTO* genotype, objective PA, and self-reported PA for both baseline and month 6, and were included in the analyses on change in PA (see Figure 1). Of these, 85.5% (430/503) and 68.0% (342/503) had 10 days of valid accelerometer wear at baseline and month 6, respectively. Mean number of valid days of accelerometer wear for these participants was 11.9 days (SD 2.1) at baseline—8.6 weekdays (SD 1.7) and 3.3 weekend days (SD 0.7)—and 10.4 days (SD 3.0) at month 6—7.7 weekdays (SD 2.3) and 2.7 weekend days (SD 1.1). This was similar for all intervention groups (data not shown for Levels 0-3).

**Table 1:** Attrition rates after 6 months by intervention level.

Group characteristics	Standard guidelines		Personalized, nongene-based advice				Personalized and gene-based advice	
	Level 0		Level 1		Level 2		Level 3	
	TT^a^	AA/AT^a^	TT	AA/AT	TT	AA/AT	TT	AA/AT
Participants, n	112	247	127	244	117	255	113	257
Dropouts, n (%)	13 (11.6)	34 (13.8)	19 (15.0)	40 (16.4)	11 (9.4)	38 (14.9)	23 (20.4)^b^	27 (10.5)^b^

^a^TT and AA/AT are the nonrisk and risk genotypes, respectively, for the fat mass- and obesity-associated (*FTO*) rs9939609.

^b^Significant difference in attrition rate between *FTO* TT and AA/AT genotypes for Level 3 participants (*P*=.01).

### Physical Activity and *FTO* Genotype

The characteristics of the 1279 participants with baseline PA data both from accelerometers and self-reports, as well as data on *FTO* genotype, are presented in Table 2. Most participants were white, 743 (58.09%) were women, and 588 (45.97%) were overweight or obese. Genotype frequency for *FTO* rs9939609 did not deviate from Hardy-Weinberg equilibrium (TT=405, TA=641, and AA=233; *P*=.48).

**Table 2:** Characteristics of the participants included in baseline analysis.

Variables		Overall (n=1279)	*FTO* ^a^risk status	
			Risk (AA/AT) (n=874)	Nonrisk (TT) (n=405)
Ethnicity (white), n (%)		1239 (96.87)	848 (97.0)	391 (96.5)
Sex (women), n (%)		743 (58.09)	520 (59.5)	223 (55.1)
Age in years, mean (SD)		40 (13)	40 (13)	40 (13)
Height (m), mean (SD)		1.71 (0.09)	1.71 (0.09)	1.72 (0.09)
Weight (kg), mean (SD)		74.8 (15.8)	75.2 (16.1)	73.9 (15.2)
BMI^b^(kg/m^2^), mean (SD)		25.5 (4.8)	25.7 (4.9)	25.0 (4.5)
Overweight (BMI 25.0-29.9 kg/m^2^), n (%)		379 (29.63)	270 (30.9)	109 (26.9)
Obese (BMI ≥30.0 kg/m^2^), n (%)		209 (16.34)	156 (17.8)	53 (13.1)
Accelerometer wear time (hours), mean (SD)		14.4 (1.1)	14.4 (1.1)	14.4 (1.0)
Number of valid days, mean (SD)		11.3 (2.4)	11.3 (2.4)	11.3 (2.4)
**Participants per season, n (%)**				
	Winter	377 (29.48)	266 (30.4)	111 (27.4)
	Spring	720 (56.29)	480 (54.9)	240 (59.3)
	Summer	99 (7.74)	73 (8.4)	26 (6.4)
	Autumn	83 (6.49)	55 (6.3)	28 (6.9)
**Participants per country, n (%)**				
	Germany	174 (13.60)	116 (13.3)	58 (14.3)
	Greece	174 (13.60)	124 (14.2)	50 (12.3)
	Ireland	178 (13.92)	123 (14.1)	55 (13.6)
	The Netherlands	214 (16.73)	148 (16.9)	66 (16.3)
	Poland	177 (13.84)	130 (14.9)	47 (11.6)
	Spain	181 (14.15)	121 (13.8)	60 (14.8)
	United Kingdom	181 (14.15)	112 (12.8)	69 (17.0)
*FTO* genotype: AA/AT/TT, n (%)		233/641/405 (18.22/50.12/31.67)	N/A^c^	N/A

^a^ *FTO*: fat mass- and obesity-associated gene.

^b^BMI: body mass index.

^c^N/A: not applicable.

**Figure 2:**
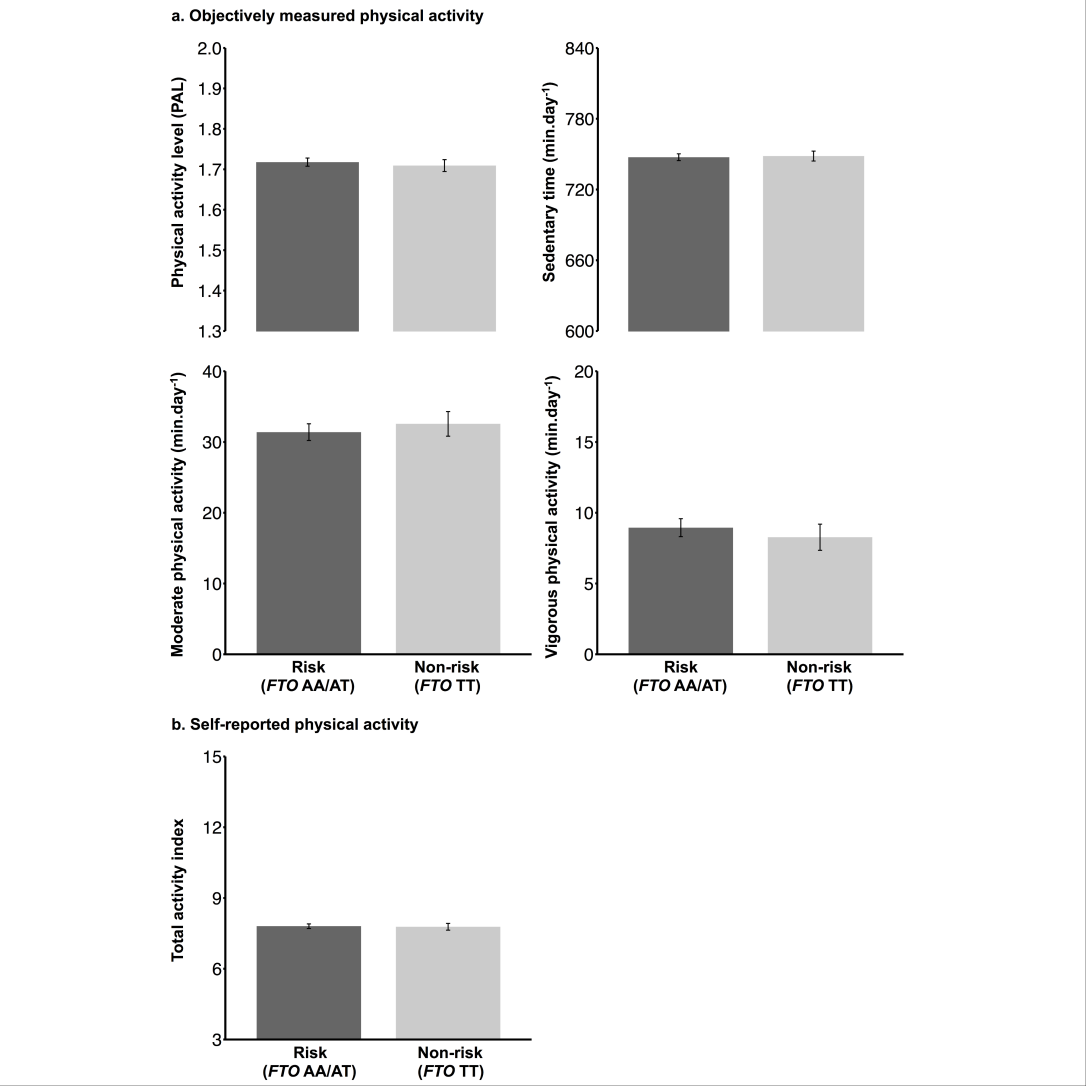
Physical activity in *FTO* rs9939609 risk (AA/AT, n=874) and nonrisk (TT, n=405) carriers. *FTO*: fat mass- and obesity-associated gene.

We found no association between objectively measured PAL (*P*=.35), moderate PA (*P*=.28), vigorous PA (*P*=.24), or sedentary time (*P*=.71) at baseline and *FTO* risk status (see Figure 2, section a). Similarly, there was no significant difference in baseline self-reported PA between risk and nonrisk carriers (*P*=.76) (see Figure 2, section b).

### Primary Analysis: Effect of Disclosing *FTO* Genotype Status on Change in Physical Activity

Table 3 displays the PA characteristics of genotyped participants advised to increase their PA at baseline, with objective PA and self-reported PA data at baseline and month 6.

**Table 3:** Changes in physical activity (PA) from baseline to month 6 for participants receiving personalized advice to increase their PA.

Variables			Disclosure (Level 3)		Nondisclosure (Levels 1 and 2)	
			*FTO* ^a^risk AA/AT (n=91), mean (SD)	*FTO* nonrisk TT (n=39), mean (SD)	*FTO* risk AA/AT (n=160), mean (SD)	*FTO* nonrisk TT (n=78), mean (SD)
**Objective PA** ^b^						
	**Daily PAL** ^c^					
		Month 0	1.64 (0.10)	1.67 (0.08)	1.68 (0.10)	1.67 (0.10)
		Month 6	1.66 (0.14)	1.70 (0.13)	1.70 (0.14)	1.70 (0.17)
	**Moderate PA (min/week)**					
		Month 0	174 (124)	209 (98)	199 (111)	189 (112)
		Month 6	206 (146)	249 (120)	218 (145)	221 (130)
	**Vigorous PA (min/week)**					
		Month 0	37 (54)	48 (67)	54 (73)	49 (64)
		Month 6	49 (76)	57 (89)	64 (93)	61 (91)
	**Sedentary time (min/week)**					
		Month 0	5449 (483)	5391 (479)	5327 (505)	5433 (485)
		Month 6	5271 (606)	5153 (449)	5172 (541)	5139 (579)
**Self-reported PA:** **total activity index**						
		Month 0	7.46 (1.49)	7.51 (1.31)	7.49 (1.37)	7.69 (1.30)
		Month 6	8.00 (1.37)	7.89 (0.99)	7.84 (1.29)	7.99 (1.44)

^a^ *FTO*: fat mass- and obesity-associated gene.

^b^PA: physical activity.

^c^PAL: physical activity level.

**Figure 3:**
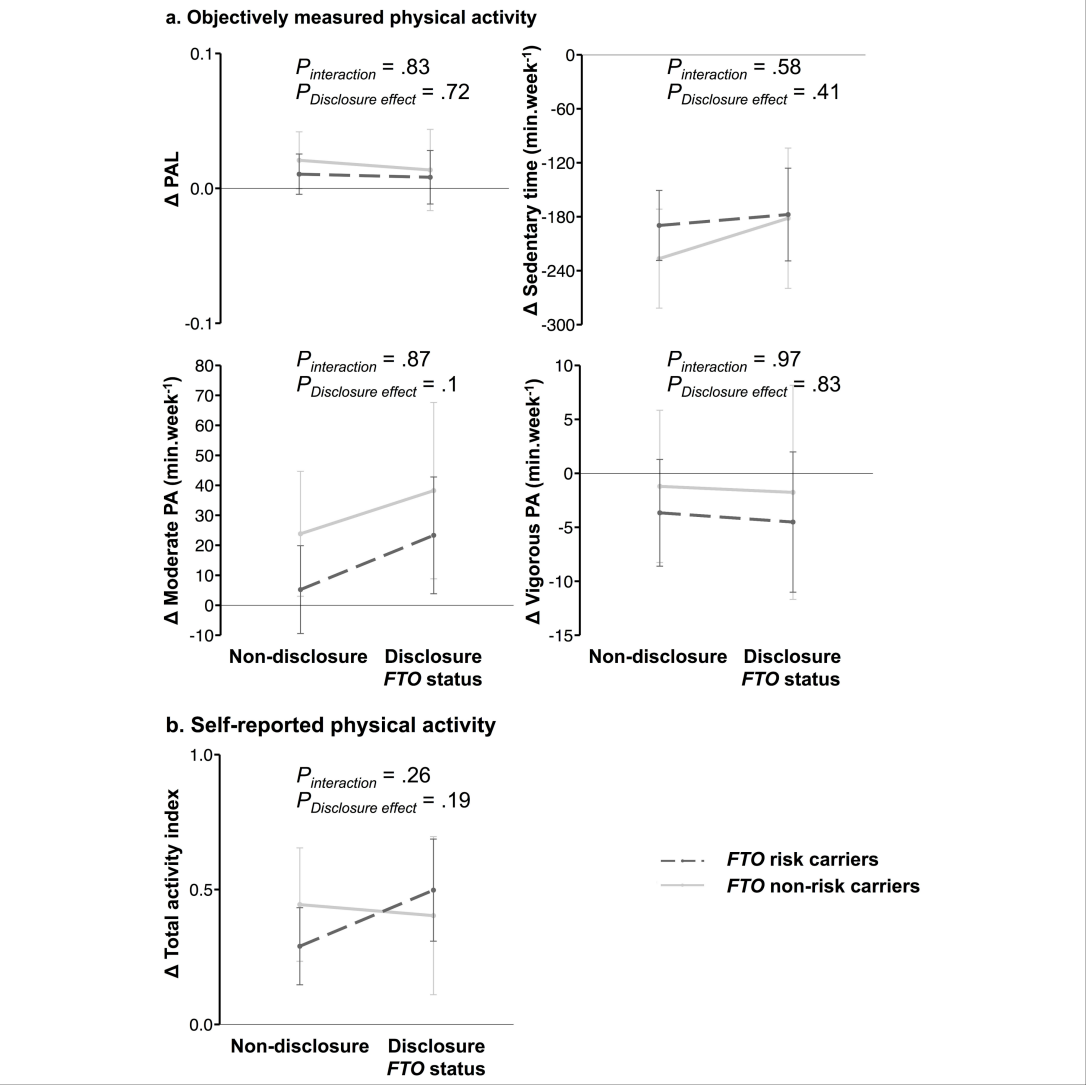
Effect of knowledge of FTO risk status on change in physical activity (PA) in risk (AA/AT) and nonrisk (TT) carriers. Nondisclosure FTO risk carriers, n=160; nondisclosure FTO nonrisk carriers, n=78; disclosure FTO risk carriers, n=91; disclosure FTO nonrisk carriers, n=39. FTO: fat mass- and obesity-associated gene.

There was no significant interaction between disclosure of genetic information and *FTO* risk status on change in objectively measured or self-reported PA (all *P*>.25); this is illustrated in Figure 3. There was also no effect of knowledge of *FTO* genotype on objectively measured or self-reported PA (all *P*>.10) (see Table 3 and Figure 3).

### Secondary Analysis: Personalized Feedback Including Disclosure of Genetic Information Compared With Standard Guidelines

Comparisons between participants in the highest level of personalization (Level 3) who were advised to increase their PA, and control participants (Level 0) who would have been advised to increase PA if they had been in a personalized group, are given in Multimedia Appendix 2. There were no significant interactions between intervention levels and *FTO* risk status on change in PA. Change in objectively measured PA did not differ significantly between Level 3 and Level 0 participants for both risk and nonrisk carriers. However, Level 3 participants, irrespective of *FTO* risk status, had greater changes in self-reported PA than Level 0 participants (see Multimedia Appendix 2).

### Sensitivity Analyses

Results and conclusions were similar when carrying out the analyses in men and women separately or after stratifying analyses by tertile of baseline PA variables (data not shown).

## Discussion

### Principal Findings

Our main findings identified that there was no association between objectively measured or self-reported PA and *FTO* risk status. To our knowledge, our study is the first to investigate the impact of *FTO* genotype-based feedback on measured change in PA in the context of a personalized lifestyle intervention. We hypothesized that knowledge of carriage of *FTO* risk would lead to an increase in PA. However, we found no evidence that disclosing such information had any positive or negative effects on PA after a 6-month intervention.

### Comparison With Previous Work

In the last decade, there has been a growing interest in personalizing lifestyle interventions using genetic tests. This has been done using DNA-based disease risk estimates, primarily in smokers or individuals at risk of certain conditions, such as Alzheimer’s disease [8]. The hope was that providing such genetic information would motivate recipients to make beneficial behavioral changes beyond what could be achieved without such information. It is unclear whether knowledge of being predisposed to a greater genetic risk of disease would promote positive behavioral change and whether knowledge of only a small genetic risk (ie, a "lower" genetic risk) predisposition would lead to counterproductive behaviors under false reassurances [33]. In their 2010 review, Marteau et al reported no effect of adding DNA-based disease risk estimates compared with a non-DNA-based approach, in terms of smoking cessation, PA, or use of medication/vitamins. A beneficial effect of DNA-based risk estimates on dietary behavior was reported, although no benefit on intention to change dietary behavior was observed [8]. Since then, Hollands et al also observed no effect of communicating DNA-based risk assessments for Crohn's disease on smoking cessation, compared with standard risk assessment [34]. Grant et al reported that diabetes genetic risk counseling did not alter self-reported motivation or adherence to a prevention program in overweight individuals at risk for diabetes [35]. Although the design of our study was different because we did not aim to recruit individuals specifically at risk of a certain disease, our results are in line with the results of most studies performed so far. Recently, Meisel et al showed that young healthy individuals receiving *FTO* feedback in their weight control advice felt more prepared to control their weight than subjects receiving weight control advice only. However, this did not translate into behavioral change [36].

Evidence in favor of disclosing genetic information is thus limited. Even the favorable findings for dietary behavior change mentioned above in the review by Marteau et al are weak [8]. They are based on only 2 studies [37,38], which did not find significant effects when each study was evaluated individually. More recently, Nielsen et al concluded that disclosing genetic information for personalized nutrition resulted in greater improvements in intake of some dietary components compared with general population-based dietary advice. In reality, this was true only for sodium intake, but not for caffeine, vitamin C, or added sugars, which were also studied. In addition, only individuals with the high-risk genotype status for the *ACE* gene reduced their sodium intake more than controls based on self-reported food intake, not on objective biomarkers of intake [39]. Similarly, Hietaranta-Luoma et al reported that personal genetic information based on *ApoE* might have positive effects on triglyceride values and waist circumference, but this was observed only in the high-risk *ε*4+ individuals [40].

Data suggest that providing genetic test results indicating a higher genetic risk does not lead to fatalism [8]. Furthermore, there is no indication that disclosing only a small genetic risk or a lower-risk test result promotes counterproductive behaviors. Similarly, in our study we found no differences in change in PA between individuals aware of their nonrisk *FTO* status and individuals aware of a risk, or not aware of their genotype. However, we did observe that the attrition rate was significantly greater among individuals informed of their nonrisk *FTO* status as compared to the other groups. Given the amount and variety of information provided to participants during the Food4Me study, it seems unlikely that this genetic information would be responsible for the higher number of dropouts. Nonetheless, this should be studied further, as it may indicate that such individuals felt the intervention was less relevant for them. Grant et al also reported that subjects receiving lower-risk genetic results showed lower intent to do exercise compared with controls, although there were no differences in terms of attendance to the diabetes prevention program [35].

Personalized feedback led to greater improvements in self-reported PA, but not objectively measured PA, compared with standard guidelines, as reported previously [19]. Discrepancies between self-reported and objectively measured PA have been noted by others. For instance, Wanner et al, in a Web-based tailored PA intervention, reported some improvements in self-reported PA after 6 weeks and 13 months of follow-up, but no differences between individuals in tailored and control groups, and no improvement in objectively measured PA for any group [41]. However, in our study we did find greater improvements in self-reported PA in tailored groups as compared with the controls. It could be that participants desired to comply with recommendations and that receiving more personalized feedback (Levels 2 and 3) increased this desire further. Furthermore, here we show that the bigger improvements in self-reported PA reported earlier are irrespective of *FTO* genotype, and are not related to knowing one’s risk status for *FTO*. Thus, it is unlikely that subjects with the high-risk variant would feel more pressured to report that they did better, compared with those with the low-risk variant. Finally, we did not observe an association between *FTO* risk and PA measured objectively or self-reported. This supports studies published thus far that have used mainly self-reported data [15,42,43].

### Strengths and Limitations

This study is the first to report the impact of disclosing information on *FTO* risk status on measured changes in PA. Our PA questionnaire has been validated against doubly labeled water and accelerometry [27,30,44], and has been used in large European cohorts before [45,46]. However, self-reports introduce large measurement error [47] and the Baecke questionnaire is no exception [48]. Thus, a strength of this study was the objective assessment of PA using triaxial accelerometers. Although accelerometers underestimate certain activities, such as cycling, swimming, or resistance training, the TracmorD model used in this study has been validated against doubly labeled water [22] and it has been shown to be reliable and accurate [49-51].

By design, we recruited individuals interested in taking part in a personalized intervention on nutrition and lifestyle, which is less representative than a European-wide survey. Nonetheless, our participants were broadly representative of the European adult population, most of whom had adequate nutrient intakes but could benefit from improved dietary choices and greater PA [52]. Given that Food4Me was an intervention that targeted multiple dietary and lifestyle behaviors, the genetic results might have also been diluted by the amount of information provided. Moreover, the genetic feedback was a positive reinforcement. Participants with the higher-risk genotype would *only* benefit more by reducing their weight or increasing their PA. It is possible that the impact would have been greater if participants had been made more aware of the links between obesity and lifelong ill health. Furthermore, genetic feedback provided by health professionals skilled in genetic counseling might have been more effective that written feedback. However, this would have been more expensive and outside the scope of this study, which was designed to test the effects of an Internet-delivered intervention. Such interventions are thought to offer considerable advantages in terms of reach, scalability, and sustainability [53]. Attrition rates (~15%) were as expected and compliance with the measurements was good, except for wearing the monitor. Only half of the participants had accelerometer data for both baseline and month 6—whereas >75% had self-reported PA data at both time points—which limited the size of the sample analyzed in the PA analyses. It is possible that wearing the monitor for 6 months was too demanding for the amount of feedback given. It may be important for future studies that participants be able to visualize their activity levels, in real time, whenever desired (eg, on an accompanying website). Improvements in activity measurement may reduce participants’ confusion and/or frustration. Having personalized coaches available, who can also operate online, may have motivated participants to wear their accelerometer and to improve their PA, although this also means extra costs. For the sole purpose of assessment, better compliance may be obtained by sending out monitors and collecting them back directly after assessment [54]. In spite of this, our sample size was acceptable, and the results did not change when looking at all self-reported PA data available.

### Conclusions

There was no added benefit of knowledge of *FTO* risk on change in PA in this intervention study. Although there were no differences in outcome measures between participants informed of a nonrisk and those informed of a risk, or those not informed of their *FTO* risk status, the nonrisk subjects were more likely to drop out of the study by 6 months. More studies are needed to confirm whether disclosure of lower-risk genetic test results has adverse effects on engagement in behavioral changes. Before that, more effort should be devoted to identify the features necessary to engage individuals, how to frame the feedback, and how to coach effectively, especially those at risk, to reduce health inequalities.

## Appendix

Multimedia Appendix 1 CONSORT-EHEALTH checklist V1.6.1 [20].

Multimedia Appendix 2 Changes from baseline in physical activity (PA) for participants who received the highest level of personalized advice (including genetic information, Level 3) and told to increase their PA, and for the controls who received standard guidelines (Level 0), but would have been advised to increase their PA if they had not been controls. Data are presented as mean (SD). *FTO*: fat mass- and obesity-associated gene.

## Abbreviations

AEE: activity energy expenditure
BMI: body mass index
BMR: basal metabolic rate
CIBERobn: Centro de Investigación Biomédica en Red-Fisiopatología de la Obesidad y Nutrición
FTO: fat mass- and obesity-associated gene
IZZ: National Food & Nutrition Institute
KASP: competitive allele-specific polymerase chain reaction
MET: metabolic equivalent
MUMC+: Maastricht University Medical Centre +
N/A: not applicable
NUTRIM: Nutrition and Translational Research in Metabolism
OR: odds ratio
PA: physical activity
PAL: physical activity level
RCT: randomized controlled trial
SNP: single nucleotide polymorphism
UCD: University College Dublin
ZIEL: Zentralinstitut für Ernährungs- und Lebensmittelforschung
