# Supplementary material for: Changes in Physical Activity Following a Genetic-Based Internet-Delivered Personalized Intervention: Randomized Controlled Trial (Food4Me)
Source: J Med Internet Res. 2016 Feb 5;18(2):e30. doi: 10.2196/jmir.5198 (PMC4761101; doi:10.2196/jmir.5198)
Supplement: Multimedia Appendix 2 [file jmir_v18i2e30_app2.pdf]

		Level 3			Level 0 (controls)				*P-values*	
**Variables**		***FTO* risk AA/AT**	***FTO* non-risk TT**		***FTO* risk AA/AT**	***FTO* non-risk TT**		Interaction Level × *FTO* risk status	Main effect Level	Main effect *FTO* risk status
		n=91	*n=39*		*n=93*	*n=42*				
Objective PA										
Daily PAL	*Month 0*	1.64 (0.10)	1.67 (0.08)		1.64 (0.10)	1.68 (0.08)		.70	.65	.47
	Month 6	1.66 (0.14)	1.70 (0.13)		1.66 (0.14)	1.70 (0.13)				
Moderate PA (min.week^{-1})	*Month 0*	174 (124)	209 (98)		167 (117)	214 (97)		.74	.90	.07
	Month 6	206 (146)	249 (120)		201 (137)	258 (153)				
Vigorous PA (min.week^{-1})	*Month 0*	37 (54)	48 (67)		44 (70)	54 (65)		.59	.68	.28
	Month 6	49 (76)	57 (89)		46 (69)	63 (74)				
Sedentary time (min.week^{-1})	*Month 0*	5449 (483)	5391 (479)		5449 (519)	5202 (440)		.87	.20	.80
	Month 6	5271 (606)	5153 (449)		5277 (516)	5034 (493)				
Self-reported PA										
Total activity index	*Month 0*	7.46 (1.49)	7.51 (1.31)		7.42 (1.40)	7.78 (1.21)		.68	**.007**	.76
	Month 6	8.00 (1.37)	7.89 (0.99)		7.50 (1.39)	7.94 (1.16)				

Data are presented as mean (SD). *FTO*, fat mass- and obesity-associated gene
